# The Anatomy of the bill Tip of Kiwi and Associated Somatosensory Regions of the Brain: Comparisons with Shorebirds

**DOI:** 10.1371/journal.pone.0080036

**Published:** 2013-11-14

**Authors:** Susan J. Cunningham, Jeremy R. Corfield, Andrew N. Iwaniuk, Isabel Castro, Maurice R. Alley, Tim R. Birkhead, Stuart Parsons

**Affiliations:** 1 Percy FitzPatrick Institute, DST/NRF Centre of Excellence, University of Cape Town, Rondebosch, South Africa; 2 Institute of Natural Resources, Massey University, Palmerston North, New Zealand; 3 Department of Neuroscience, University of Lethbridge, Lethbridge, Alberta, Canada; 4 Institute of Veterinary, Animal and Biomedical Sciences, Massey University, Palmerston North, New Zealand; 5 Department of Animal and Plant Sciences, University of Sheffield, Western Bank, Sheffield, United Kingdom; 6 School of Biological Sciences, University of Auckland, Auckland, New Zealand; Claremont Colleges, United States of America

## Abstract

Three families of probe-foraging birds, Scolopacidae (sandpipers and snipes), Apterygidae (kiwi), and Threskiornithidae (ibises, including spoonbills) have independently evolved long, narrow bills containing clusters of vibration-sensitive mechanoreceptors (Herbst corpuscles) within pits in the bill-tip. These ‘bill-tip organs’ allow birds to detect buried or submerged prey via substrate-borne vibrations and/or interstitial pressure gradients. Shorebirds, kiwi and ibises are only distantly related, with the phylogenetic divide between kiwi and the other two taxa being particularly deep. We compared the bill-tip structure and associated somatosensory regions in the brains of kiwi and shorebirds to understand the degree of convergence of these systems between the two taxa. For comparison, we also included data from other taxa including waterfowl (Anatidae) and parrots (Psittaculidae and Cacatuidae), non-apterygid ratites, and other probe-foraging and non probe-foraging birds including non-scolopacid shorebirds (Charadriidae, Haematopodidae, Recurvirostridae and Sternidae). We show that the bill-tip organ structure was broadly similar between the Apterygidae and Scolopacidae, however some inter-specific variation was found in the number, shape and orientation of sensory pits between the two groups. Kiwi, scolopacid shorebirds, waterfowl and parrots all shared hypertrophy or near-hypertrophy of the principal sensory trigeminal nucleus. Hypertrophy of the nucleus basorostralis, however, occurred only in waterfowl, kiwi, three of the scolopacid species examined and a species of oystercatcher (Charadriiformes: Haematopodidae). Hypertrophy of the principal sensory trigeminal nucleus in kiwi, Scolopacidae, and other tactile specialists appears to have co-evolved alongside bill-tip specializations, whereas hypertrophy of nucleus basorostralis may be influenced to a greater extent by other sensory inputs. We suggest that similarities between kiwi and scolopacid bill-tip organs and associated somatosensory brain regions are likely a result of similar ecological selective pressures, with inter-specific variations reflecting finer-scale niche differentiation.

## Introduction

One of the most diverse characteristics of birds is the bill. Bills are uniquely adapted to carry out a multitude of functions, including preening, manipulating objects, fighting, courtship, feeding young and most importantly, the acquisition and handling of food. The size and shape of a bill is often directly related to species-specific feeding behaviors. The avian bill is also a complex sensory structure, containing at least two types of mechanoreceptors: Grandry corpuscles, which detect velocity; and Herbst corpuscles, which detect pressure [1-3]. At least five clades of birds have bills containing high concentrations of these mechanoreceptors: waterfowl (Anseriformes), parrots (Psittaciformes), shorebirds (Charadriiformes, specifically Scolopacidae), ibises and spoonbills (Ciconiiformes, Threskiornithidae) and kiwi (Apterygiformes, Apterygidae) [1,4-16]. In Scolopacidae, Threskiornithidae, and Apterygidae, the mechanoreceptors are clustered into pits (‘sensory pits’) within the bones of the bill tip [11,13,17-19], and each individual cluster may act as a functional unit [20]. In parrots, the mechanoreceptors are arranged within touch papillae embedded in holes within the keratin rhamphotheca [16,21]. Waterfowl possess parrot-like papillae that emerge from the deep dermis behind the keratinaceous nail of the bill tip, as well as sensory pits containing mechanoreceptors in the distal portions of the bill bones [1,4]. Whether configured within touch papillae or sensory pits, these dense clusters of mechanoreceptors at the bill tips are commonly referred to as a ‘bill-tip organs’ (e.g. [1,7]).

The numbers, distribution pattern and location of mechanoreceptors varies among species and, as with bill morphology, corresponds to specific feeding strategies. For example, mallards (Anseriformes: *Anas platyrhynchos*) have mechanoreceptors that are concentrated in the tip and ridges of the bill, as well as on the tongue, all of which facilitate the detection, recognition, and transportation of food in the mouth [4,22]. In parrots, the bill-tip organ seems to be associated more with an enhanced ability to manipulate objects, with mechanoreceptors found at the tip and along the inner ventral edges of the rhamphotheca, as well as in the tongue [16,21,23]. The remaining taxa, the scolopacids, ibises and kiwi, all share a common foraging strategy: bill probing for invertebrates and small vertebrates buried beneath the substrate, or sweeping for prey in the water column in the case of spoonbills and aquatic-foraging ibises. All three groups have evolved elongated bills with clusters of mechanoreceptors concentrated in the bill tip [7,9,10,13,15,20,24-26]. Their specialized bill tips allow these species to detect buried/submerged prey by picking up vibrotactile and/or pressure cues in the substrate [7,9,10,13-15,20]. The three groups are not closely related, with the paleognathous kiwi being separated by a particularly deep evolutionary divide from the neognathous shorebirds and ibises [27,28]. This suggests the bill-tip organs of kiwi have evolved independently of those in ibises or scolopacids. Details of the extent to which the bill morphology and organization of mechanoreceptors in kiwi bills is similar to those of the other two groups are not known.

The mechanoreceptors of the bill tip represent the beginning of a somatosensory pathway that projects via the trigeminal nerve (nV) to the principal sensory trigeminal nucleus (PrV,[29-31]), found dorsal to the root of nV in the anterior brainstem [29]. The PrV then relays information to the telencephalic sensory end-station, the nucleus basorostralis (Bas), which contains a somatotopic map of the bill tip, as well as other parts of the body [31-35]. Both the size and cytoarchitecture of the trigeminal system are highly variable among species [6,8,30,36,37], and these differences likely reflect both the relative importance of tactile information in foraging behavior, and the distribution and number of mechanoreceptors in the bill ([8], and see above). For example, of the taxa with specialized bill tips, waterfowl, bill-probing shorebirds, and parrots all have relatively large PrV volumes compared with other birds (e.g., Galliformes) [8]. An increase in the relative size of PrV may therefore be associated with the use of the bill in tactile-based food acquisition, in a similar fashion to the enlargement of auditory regions and acoustic prey localization in owls [38-40]. Such enlargements are in keeping with the ‘principle of proper mass’, which predicts that the relative size of a neural structure is correlated with the complexity of the associated behavioral or, in this case, sensory capability of the animal [41]. Although both PrV and Bas are relatively large and well-defined in kiwi [12], there has been no detailed analysis of this enlargement or how it compares with that of shorebirds and other tactile specialists. 

Kiwi appear to be uniquely adapted to function in a nocturnal ground-dwelling niche, with changes to both the peripheral and central nervous systems. Kiwi have enlargements to the olfactory system and other specific regions in the telencephalon [42-44], a specialized auditory system [45-47], developed facial bristles [48] and a highly regressed visual system [12,49]. It would therefore be of interest to further examine the tactile system of kiwi to determine if, like many of their other sensory systems, it has undergone changes associated with their unique niche. The aims of this study are therefore to examine the bill-tips of kiwi and compare them with shorebirds, which have a well characterized bill-tip organ, and also to describe and measure PrV and Bas to determine if kiwi have an enlarged trigeminal system similar to some shorebirds, ducks and parrots. 

## Methods

### Ethical Statement

All specimens used in the study were provided to us postmortem by conservation authorities, wildlife veterinarians, hunters and farmers and were not killed specifically for this study, thus no ethics approvals were required to undertake this research. North Island brown kiwi (*Apteryx mantelli*), bar-tailed godwits (*Limosa lapponica*), South Island oystercatchers (*Haematopus finschi*), black-winged stilts (*Himantopus himantopus*) and masked lapwings (*Vanellus miles*) are protected in New Zealand and permission to use these specimens for research was obtained under permits NO-16732-FAU, NO-18095-DOA, WC-17552-DOA, WE-333-RES, WA-24648-RES, NO-27881-RES from the New Zealand Department of Conservation. 

### Specimens

North Island brown kiwi, bar-tailed godwits, South Island oystercatchers, black-winged stilts and masked lapwings were obtained from Northland, New Zealand and were obtained directly from the Department of Conservation. Additional North Island brown kiwi and bar-tailed godwit specimens were obtained from the National Wildlife Mortality Database, Massey University, where they had been archived in formalin for research purposes following necropsy. Eurasian woodcocks (*Scolopax rusticola*) were provided by A. Hoodless and M. Swann and were obtained from West Woodyates, Dorset, United Kingdom. Tinamou brains (*Nothura darwinii*, *Rhynchotus rufescens* and *Tinamus major peruvianus*) were provided by P. Brennan and ostrich (*Struthio camelus*) and emu (*Dromaius novaehollandiae*) brains were obtained from farmed birds owned by Northland Ostrich and Emu Ltd., New Zealand. A Hoodless, M. Swann, and P. Brennan all had the required permits, licensing and ethics approval to obtain the woodcock and tinamou specimens. All other species were obtained from local farmers and hunters in New Zealand where these animals are regularly shot by landowners because they are either pests or game birds. We did not require any permits or licensing to obtain postmortem specimens from hunters or farmers in New Zealand. Permits are only required if the birds were shot directly for the study, which is not the case in this study. Species were named according to Gill & Donsker [50].

To assess the magnitude of any enlargement of the PrV and Bas of kiwi relative to other birds, we compared the measurements of these regions with both bill-probing shorebirds, and a number of non probe-foraging species, drawn from a wide range of avian families (see Table 1 and Table S1). 

**Table 1:** List of the species surveyed, sample size and volumes (in mm^3^) of the brain, telencephalon (Tel), hindbrain (HB), principal sensory trigeminal nucleus (PrV), and nucleus basorostralis (Bas).

**Order**	**Common name**	**Species**	**n**	**Brain**	**Tel**	**HB**	**PrV**	**Bas**
Anseriformes	Paradise shelduck	*Tadorna variegata*	3	4157.38	2689.60	322.00	8.38	46.03
Charadriiformes	Bar-tailed godwit	*Limosa lapponica*	2	2417.27	1563.49	158.37	5.18	29.96
	Masked lapwing	*Vanellus miles*	3	2067.13	1185.57	137.86	1.02	10.06
	Eurasian woodcock	*Scolopax rusticola*	2	2325.59	1431.58	203.82	7.84	14.80
	South Island oystercatcher	*Haematopus finschi*	2	2917.70	1757.44	217.16	2.81	33.29
	Black-winged stilt	*Himantopus himantopus*	2	1678.49	998.82	107.60	1.07	9.13
Columbiformes	Rock pigeon	*Columba livia*	3	1705.66	893.05	123.58	0.46	5.02
Galliformes	Peafowl	*Pavo cristatus*	3	4559.48	2665.26	349.16	1.41	8.28
	Turkey	*Meleagris gallopavo*	3	5274.02	2914.66	417.37	1.28	10.13
	California Quail	*Callipepla californic*	1	990.37	544.18	78.45	0.32	2.44
Passeriformes	Australian magpie	*Gymnorhina tibicen*	4	4664.24	3443.98	154.95	0.45	5.39
Psittaciformes	Eastern rosella	*Platycercus eximius*	4	2685.74	2032.15	104.52	2.44	9.41
Rallidae	Pukeko	*Porphyrio porphyrio melanotus*	3	4186.05	2771.03	254.90	2.33	18.76
Struthioniformes	Emu	*Dromaius novaehollandiae*	1	21829.88	13695.99	1417.55	3.39	107.53
	Ostrich	*Struthio camelus*	1	27006.26	17984.78	1712.31	9.84	80.10
Apterygiformes	North Island brown kiwi	*Apteryx mantelli*	2	5298.95	4267.72	237.13	6.98	41.32
Tinamiformes	Darwin’s nothura	*Nothura darwinii*	2	1482.37	809.09	125.99	0.37	5.27
	Great tinamous	*Tinamus major*	1	2242.13	1221.88	184.05	0.93	7.47
	Red-winged tinamou	*Rhynchotus rufescens rufescens*	1	3013.88	1704.73	221.29	0.97	9.72

The Charadriiformes taxa included in this study were chosen to represent species that forage in a range of habitats and also to include some that are known to have a specialized sensory bill-tip organ and others that are not. Bar-tailed godwits and Eurasian woodcocks both appear to have sensory bill-tip organs to detect invertebrates beneath the ground by remote sensing of vibrations/pressure gradients. However, these species differ in habitat use, with godwits foraging in mudflats, grassland and marshes and woodcocks in woodland and wet grassland habitats, the latter habitat being mainly used at night [17,18,51-53]. In contrast, both South Island oystercatchers and black-winged stilts detect their buried and/or underwater prey by direct touch (direct contact of the prey-item by the bill tips, as opposed to initial detection of pressure/vibratory cues at some distance from the prey) in inter- tidal flats and pools [53-56]. Oystercatchers will also frequently take and open the shells of mollusks from the surface and this is probably done using vision rather than touch [55,56]. Finally, masked lapwings have a short bill with which they do not probe, but rather peck at prey on the ground that are likely to have been located visually [57].

### Bill processing and analysis

X-ray micro computed-tomography (micro CT) was used to image the bill tip of two adult male and one adult female North Island brown kiwi, one adult female bar-tailed godwit, one adult South Island oystercatcher, one black-winged stilt, and one Eurasian woodcock. All bills were immersion-fixed in 4% paraformaldehyde (PFA) in phosphate buffered saline (PBS) for at least 1 week prior to scanning. The tips of the bills were trimmed from the rest of the carcass and wrapped in cling film to keep the upper and lower pieces in the correct (natural) position with respect to one another. The bills from the kiwi, godwit, oystercatcher and stilt were mounted vertically in a Skyscan-1172 micro CT scanner and scanned at a 17.3 µm resolution. The bill tip of a woodcock was scanned using a Scanco Medical AG µCT 35 scanner at a 12 µm resolution. Three-dimensional models of the bill-tips of all species were reconstructed in AMIRA (v5.2, Visage Imaging) using the micro CT images. Measurements of features of the bill-tips were made from raw micro CT images using image analysis software ImageJ [58]. Models and raw images were used to describe the morphology of the premaxilla and dentary bones in detail (the distal-most bones of the upper and lower bill, respectively) and to count any sensory pits. For kiwi, the description is based on all three specimens used for micro CT, and measurements given are averages of the three birds (± 1 SD). ‘Proximal’ is used to describe regions of the bill closer to the head, ‘distal’ to describe regions closer to the bill-tip.

Tissues for bill histology were obtained from five North Island brown kiwi (three adult males, one juvenile male and one juvenile female) and one adult female bar-tailed godwit, but were not available for other species used in this study. The first 14 mm of the bill-tips of the godwit and four of the kiwi were trimmed from the carcasses, then upper and lower bill-tip trimmings were split longitudinally for sectioning in the sagittal plane. The bill-tip of the 5^th^ kiwi (an adult male) was trimmed coronally at 3, 6 and 9 mm from the tip of the upper bill rhamphotheca and 2 mm from the tip of the lower bill rhamphotheca (corresponding to the 9 mm upper bill trimming), for sectioning in the coronal plane. We also attempted coronal sections from more proximal portions of the bar-tailed godwit bill, but these were unsuccessful due to the fragile nature of the sample. The keratin rhamphotheca of each specimen was softened following [59] and the trimmed pieces decalcified using neutral EDTA [60], embedded in paraffin, and sectioned at 3 µm thickness. The sections were stained with haematoxylin and eosin and Masson’s trichrome [59]. We measured width, length and numbers of Herbst corpuscles per sensory pit from digital photomicrographs of sagittal sections from each specimen. Measurements were made using ImageJ and are given as means ± 1 SD. We made drawings from the coronal sections of the adult male brown kiwi bill to show the positions of major nerves and blood vessels. 

### Brain processing and analysis

All brains were immersion-fixed in 4% PFA in PBS. Prior to sectioning, brains were cryoprotected in 30% sucrose in 0.1 M PBS until they sank (usually between 3 days to 1 week) and embedded in a solution of 15% gelatin with 30% sucrose and black fabric dye (to darken the gelatin solution). To align the tissue sections after processing, brains were embedded using a custom-made mould so that fiduciary points could be placed in the gelatin (see 43). The block was sectioned on a sliding freezing microtome at 50 µm thickness in the sagittal plane. For each species, except for emu and ostrich (every fourth), every second section was mounted serially onto subbed slides, stained with cresyl violet, dehydrated and coverslipped with DePeX from xylene. Sections and fiduciary points in the surrounding gelatin were imaged using a Leica stereomicroscope. In addition, photomicrographs of every second section were taken throughout the rostrocaudal extent of Bas and PrV. 

We measured the post-processing volumes of the whole brain, telencephalon, Bas, PrV and hindbrain. We defined the hindbrain as rostral border of the isthmus to the caudal border of pseudorhombomere 11 (as described in [61]), less the cerebellum. PrV and Bas were identified using the same criteria as previous studies [5,8,29,32,33,62,63]. The digital photomicrographs were loaded into AMIRA so that each brain region could be selected and exported as a series of TIFF files. In these, a given brain region is filled in black against a white background. These TIFF stacks were then used for volumetric estimates of each region using ImageJ. Each image was then analyzed to obtain the cross-sectional area of the brain object. The cross-sectional areas were added for each brain region and then multiplied by the slice thickness and the number of sections between stack slices. 

In addition to the volumetric data obtained from these brains, we also included data from a number of species included in the studies of [37] and [8], (Table S1). The addition of this data broadened the sample of species, including non-probe foraging birds and additional ratite taxa, for comparison with kiwi and shorebirds of interest. 

To account for allometric effects on brain region volume, all measurements were examined relative to the total brain, telencephalon and hindbrain volume. Data were log_10_ transformed prior to analyses and the volume of each brain region was compared with brain volume minus the volume of the region of interest. We performed least squares linear regressions using each of the dependent variables against the scaling variables outlined above. We then calculated 90 and 95% confidence intervals for these regression lines and screened for significant outliers by examining jackknife distances as calculated in JMP v. 5.1.2 (SAS Institute). We used 90 and 95% CIs as thresholds to assess whether PrV and Bas could be considered hyper- or hypo-trophied, relative to the volume of other brain structures in a similar fashion to other studies of brain volumetrics (e.g., [43,64]).

In addition to analyses of species as independent data points, we also accounted for phylogenetic effects. We first constructed phylogenetic trees of inter-ordinal relationships based on Livezey and Zusi [28] and Hackett, et al. [27] in Mesquite [65]. Resolution within orders was provided by additional sources [66-70]. Because we reconstructed the phylogeny of all species from multiple sources, we used an arbitrary branch length model, which we then used to construct ‘phylogeny-corrected’ confidence intervals [71,72] using the PDAP: PDTREE module of Mesquite [73]. Outliers and the confidence intervals themselves were not different between the two phylogenies, so for simplicity, we only present those based on Hackett, et al. [27]. 

### 3D reconstructions

The overall morphology of kiwi brains differs from that of other birds in several respects [43,44,49,74]. We used AMIRA to construct 3D models of two brain regions that process tactile information to visualize their morphology and compare this to other species. Firstly, Bas is somatotopically organized and species differences occur in the proportion of Bas dedicated to each body region [31-35]. Differences in Bas morphology might reflect species differences in somatotopic representations. Secondly, the hyperpallium, in particular the rostral Wulst, is somatosensory in nature, receiving projections from the bill via the thalamus in some species [75-78]. Bar-tailed godwits and kiwi share a caudal displacement of the hyperpallium which could be associated with their bill tip specialization or possibly with a reduction to the visual system [12,43,44]. A larger comparative study of the placement of the hyperpallium may help to reveal the significance of this feature. In addition, we also included the striatopallidal complex (SPC; mediale and laterale) in the models for orientation purposes (see 43).

## Results

### External morphology and structure of the keratin coat

**Figure 1:**
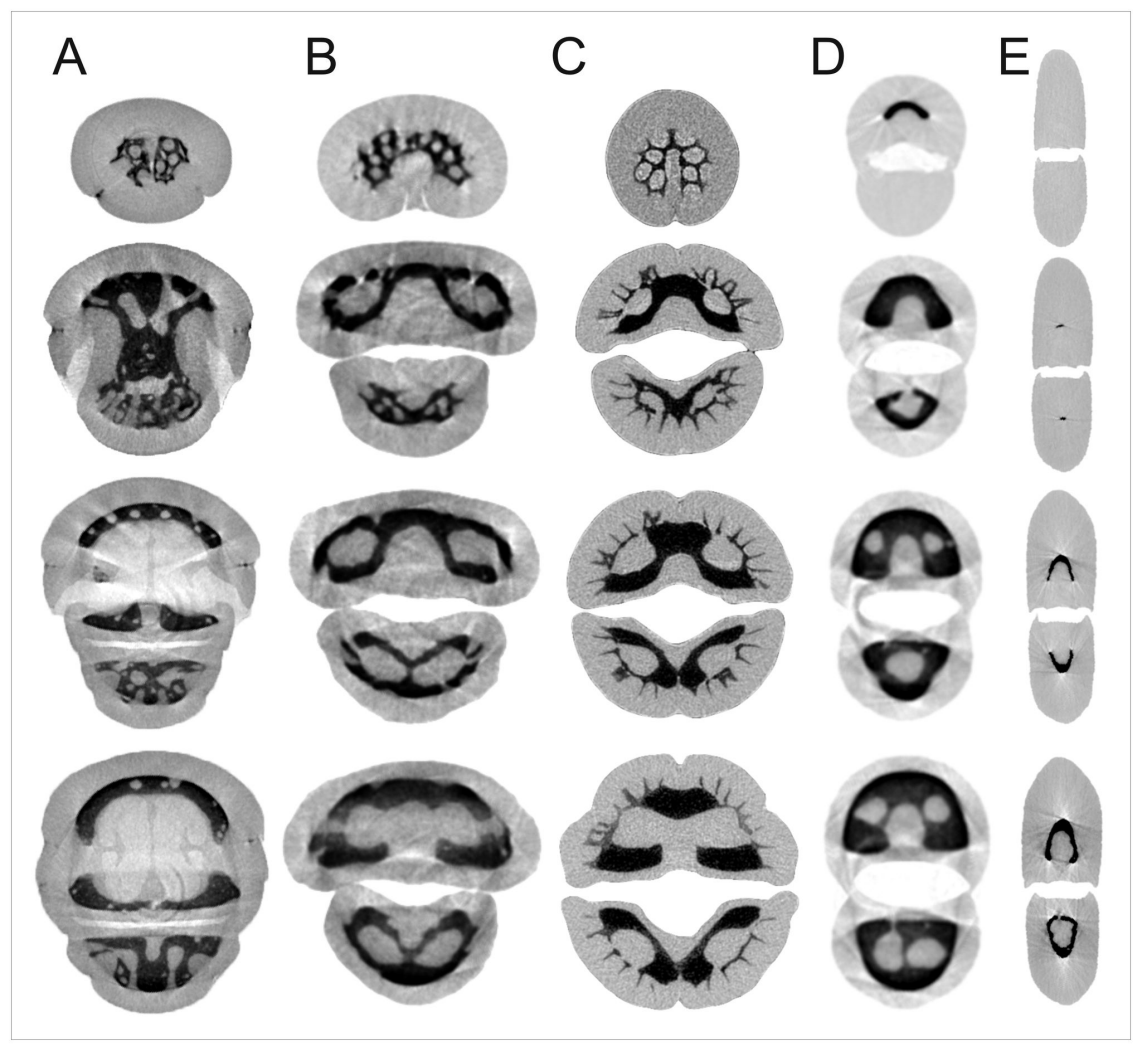
Micro CT coronal sections through bill-tips of five probe-foraging bird species. A: North Island brown kiwi (*Apteryx mantelli*), B: bar-tailed godwit (*Limosa lapponica*), C: Eurasian woodcock (*Scolopax rusticola*), D: black-winged stilt (*Himantopus himantopus*), and E: South Island oystercatcher (*Haematopus finschi*). Consecutive slices are 3 mm apart. Mid-grey areas = keratin and soft tissue, dark-grey to black areas = bone. Scale bar = 2 mm.

In all species examined, except the oystercatcher, the upper bill tip overlapped the lower bill tip. The extent of overlap, measured from the tip of the upper rhamphotheca to the tip of the lower, was greatest in kiwi and least in the stilt (Table 2). In addition, the rhamphotheca extended distally beyond the tips of the premaxilla and dentary bones to differing extents in all species, but was much longer in the stilt and the oystercatcher (Table 2). The oystercatcher bill was also flattened mediolaterally, whereas the bills of the other four species were essentially circular in cross-section (Figure 1). Kiwi differed from all other species in that the nares were positioned near the bill-tip and therefore included in the trimmed sample. The nares of kiwi were downward-facing and shielded dorsally by ‘curtains’ of keratin. 

**Table 2:** Aspects of the bill morphology of five species based on µCT scans.

**Species**	**n**	Overlap of lower by upper beak (mm)*	**Extention of rhamphotheca distal of premaxilla tip (mm)**	**Extention of rhamphotheca distal of dentary tip (mm)**	**Extent of premaxillary symphysis (mm)**
North Island brown kiwi	3	55.57 ± 0.74	1.27 ± 0.36	0.62 ± 0.29	4.2 ± 0.2
Eurasian woodcock	2	2.87 ± 0.13	0.9	0.64	11.8
Bar-tailed godwit	1	2.27	1.42	1.69	9.3
Black-winged stilt	1	1.45	4.34	4.15	> 11.7*
South Island oystercatcher	1	0	9.84	9.57	> 6.3*

* symphysis occurred proximal of the cut edge of the sample

### Morphology of the premaxilla

The premaxilla bone in birds tends to be three-pronged, with the three pieces (rami) fusing distally, creating a solid tip to the bill that may vary in size in proportion to the rest of the bill. In many probe-foraging species, sensory pits are found on the surfaces of this fused area and its size may therefore be important. Fusion of the three parts of the premaxilla occurred distal of the cut edges of the kiwi, godwit and woodcock samples (Table 2). In the godwit and woodcock the outer edges of the dorsal ramus fused with the outer edges of the ventral rami at 9.3 mm (godwit) and 11.8 mm (woodcock) from the premaxilla tip. The inner edges of the ventral rami then fused with the dorsal ramus at 8.9 mm (godwit) and 9.0 mm (woodcock) from the tip of the premaxilla, creating two large, parallel channels measuring 0.7 x 0.5 mm (left) and 0.6 x 0.5 mm (right) in the godwit, and 0.9 x 0.7 mm each in the woodcock (Figure 1B, C). These channels narrowed distally in both birds, and at 1.3 mm (godwit) and 1.9 mm (woodcock) from the tip of the premaxilla, they began to separate into multiple, forward facing sensory pits.

**Figure 2:**
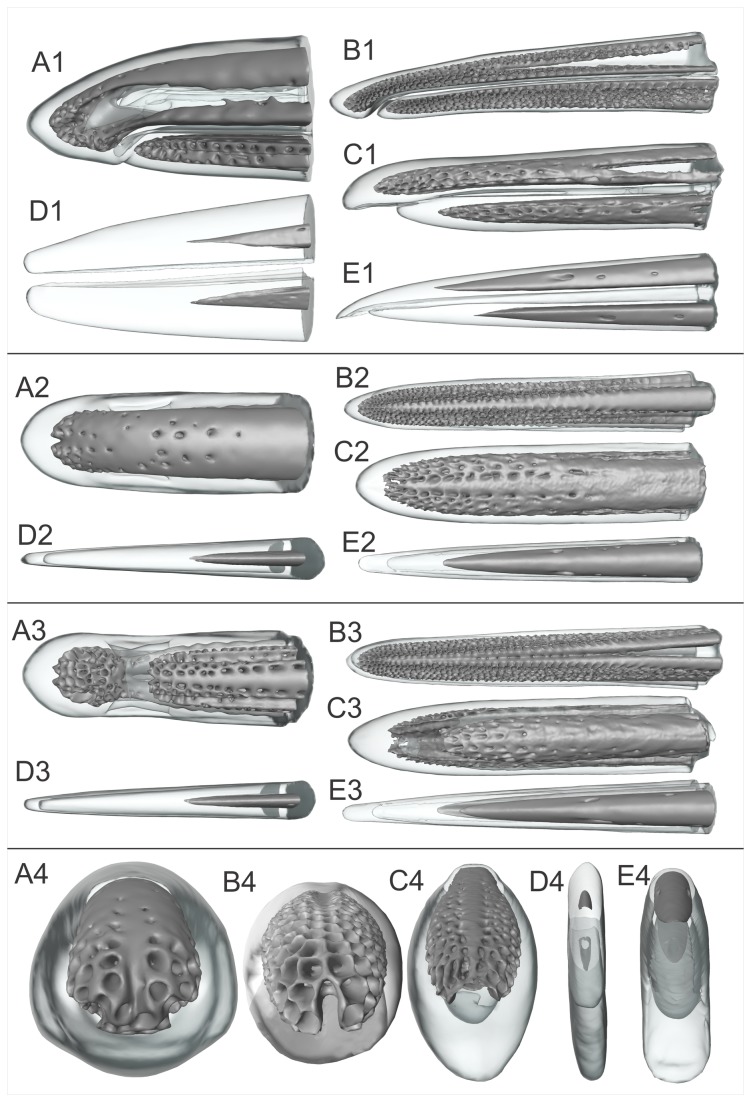
3D reconstructions of the bill-tips of five probe-foraging bird species. A1-4: the North Island brown kiwi (*Apteryx mantelli*), B1-4: Eurasian woodcock (*Scolopax rusticola*), C1-4: bar-tailed godwit (*Limosa lapponica*), D1-4: South Island oystercatcher (*Haematopus finschi*), and E1-4: black-winged stilt (*Himantopus himantopus*). Panel one shows a lateral view, panel two a dorsal view, panel three a ventral view, and panel four a rostral view. The dark grey structure represents the bone and the transparent structure the keratin.

**Figure 3:**
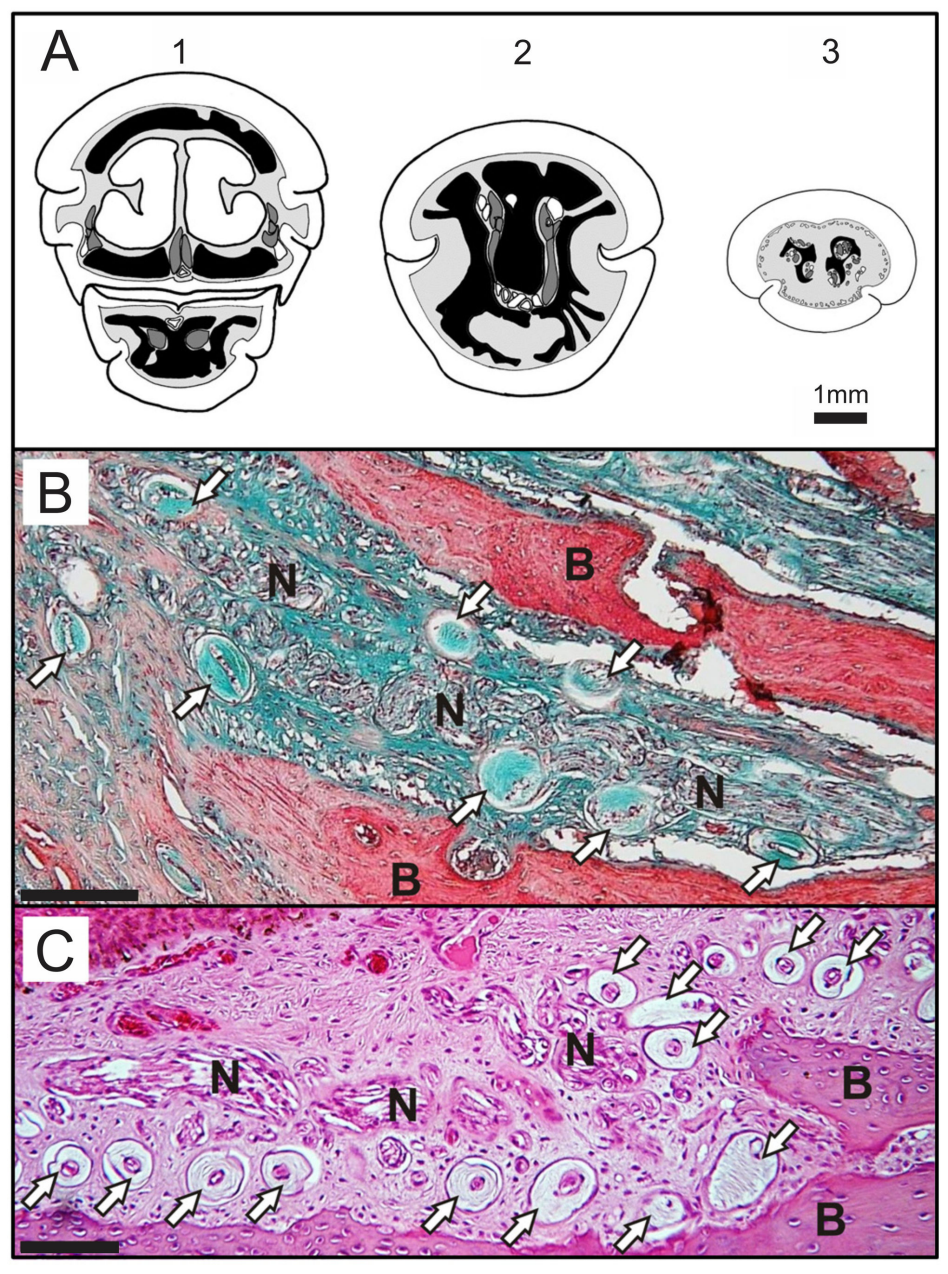
Histological sections of North Island brown kiwi (*Apteryx mantelli*) and bar-tailed godwit (*Limosa lapponica*) bill-tips. A: Diagram of three coronal sections through a North Island brown kiwi bill, taken at: (1) 9 mm from the tip of the upper bill rhamphotheca (showing upper bill above, lower bill beneath); (2) 6 mm from tip of the upper bill rhamphotheca , sectioned through the sensory pad area forward of the tip of the rhamphotheca of the lower bill); (3) 3 mm from the tip of the upper bill rhamphotheca. Black areas represent the premaxilla and dentary bones. Dark grey shaded areas represent cross sections through the major nerves. Areas of soft tissue are shaded pale grey, the keratin and major blood vessels are left white. Herbst corpuscles (in 3) are white, with a central black line to represent the nerve axon. Bold lines indicate the outer surface of the keratin layer, finer lines indicate the junction between the dermal and keratin layers and the outlines of major blood vessels, nerves, and Herbst corpuscles. In (1), the premaxilla is perforated by the two nasal passages, bordered with bold lines and colored white. B: Sagittal section through a sensory pit in the North Island brown kiwi premaxilla, stained with Masson’s trichrome and C: a sensory pit in the bar-tailed godwit dentary, stained with haematoxylin and eosin. Abbreviations: N: nerves, B: bone. Examples of Herbst corpuscles are highlighted with white arrows. Scale bars = 100 µm.

Fusion of the premaxilla was achieved differently in kiwi, perhaps due to the presence of the narial openings near the tip of the bill (4.3 ± 0.5 mm, extending to 7.1 ± 0.4 mm, from the tip of the premaxilla bone; Figures 2 A1, 2, 3) and the consequent necessity for the premaxilla to support the olfactory canals along much of its length (visible in Figure 1A, Figure 3 A1). The ventral rami fused towards the distal end of the narial openings (4.7 ± 0.4 mm proximal of the premaxilla tip), and a ventral process of the dorsal ramus with the ventral rami at 4.2 ± 0.2 mm proximal of the premaxilla tip. Fusion of the premaxilla in kiwi created a much more complex pattern of channels in the bone than in the woodcock and godwit, as described below.

Proximal to the point of fusion, the dorsal ramus of the kiwi premaxilla carried two narrow, longitudinal channels (‘dorsal channels’, Figure 1A), which opened into a double row of sensory pits and in one individual, an additional scatter of pits lateral to the double row (Figure 2, A2). These two channels did not extend proximally beyond the most proximal sensory pit. Distally, however, they extended into the bill tip, past the point of fusion. The premaxilliary fusion left two further large circular channels, one below the other, (‘central’ 0.7 ± 0.1 mm in diameter, ‘ventral’ 0.5 ± 0.1 mm in diameter) for nerve and blood vessel entry to the distal bill-tip organ. At 3.8 ± 0.2 mm proximal from the premaxilla tip, the central channel split into two branches, which fused distally with the left and right dorsal channels, forming two long, narrow slots. Distal from this point, these two slots lengthened vertically and fused with the ventral channel, creating a U-shaped cavity within the premaxilla bone. Histological sections showed that each of the arms of the ‘U’ contained large, laterally flattened nerve branches; the bottom of the ‘U’ contained numerous blood vessels (Figure 3, A2). The club shaped block of bone in the centre of the cavity lengthened distally to meet the bone of the sensory pad area at 3.0 ± 0.3 mm proximal to the premaxilla tip, dividing the cavity into two vertically elongated slots measuring 1.9 ± 0.1 x 0.3 ± 0.1 mm (left) and 1.9 ± 0.2 x 0.3 ± 0.1 mm (right); which began to branch off into individual sensory pits at 2.5 ± 0.2 mm from the premaxilla tip (Figure 1, A; Figure 3, A3). Proximal to premaxilliary fusion, histological sections revealed three nerve bundles within the premaxilla, positioned below the olfactory canals in lateral left and right, and medial positions (Figure 3, A1). 

The premaxilla of the stilt, like that of the woodcock and godwit, contained two parallel, circular channels (0.5 x 0.4 mm left, 0.4 x 0.4 mm right at 11.9 mm proximal of the bill tip, Figure 1D) that narrowed and eventually emerged as neurovascular foramina in the dorsolateral surface of the bone between 6.1 and 4.8 mm proximal of the tip (left); and between 5.6 and 4.7 mm proximal from the tip (right). The oystercatcher premaxilla was shaped like an inverted ‘U’ at the cut surface of the sample (6.3 mm proximal of the premaxilla tip) and contained no channels or pits. The bone maintained its shape, but gradually tapered towards a fine tip at 9.84 mm proximal to the tip of the rhamphotheca (Figure 1E, Figure 2, D1). 

### Morphology of the dentary

In kiwi, the dentary bone contained a deep, dorsomedial groove that carried an artery (Figure 3 A1), whereas in woodcock and godwit the dentary was u-shaped (Figure 1B and C), in the stilt semi-circular (Figure 1D), and in the oystercatcher formed a hollow tube at the cut edge of the sample, which opened into a deep u-shape 6 mm distal from the tip of the bone (Figure 1E). 

In kiwi, godwit, woodcock, and stilt, the dentary bone contained two parallel, wide channels that tapered towards the tip. In kiwi, godwit and woodcock, these channels separated near the tip into numerous sensory pits. Histological sections showed that each channel carried a large nerve in kiwi (presumably a branch of the mandibular ramus of the trigeminal nerve; Figure 3, A1). In the stilt, the two channels merged together 6 mm from the dentary tip (Figure 1D). At 2.6 mm distal from the stilt bill-tip, this central channel opened broadly (0.2 mm) to the dorsal surface of the dentary, becoming a deep groove in the bone.

### Sensory pits: Appearance, number and histology

Three of the five species examined by micro CT (woodcock, kiwi and godwit) possessed large numbers of sensory pits in both the premaxilla and dentary bones (Figure 2). Numbers of sensory pits in the most distal 15 mm of the bill-tip, and shape and general orientation of the sensory pits are summarized in Table 3. 

**Table 3:** Morphology and numbers of sensory pits in the North Island brown kiwi, Eurasian woodcock and bar-tailed godwit.

**Species**	**Bill bone**	**Sensory pit shape**	**Sensory pit orientation**	**Sensory pit size: width at surface x depth (mm) (*n pits, n individuals*)**	**Number of sensory pits**		
					**0-5 mm**	**5-10 mm**	**10 -15 mm**
North Island brown kiwi	Premaxilla	Oval in cross-section, tapering with depth. Broad, ovoid perforations at the base communicate with underlying nerve channels.	Backward-facing proximal of the premaxillary symphysis, outwards-facing distal of the nares, forward-facing at bill-tips. Face downward and forward in sensory pad region.	0.35 ± 0.07 x 0.56 ± 0.18 *(57,3)*	113	15	5
	Dentary	As above.	Ventral, lateral and dorsal sensory pits outward-facing; forward-facing at bill tips.	0.36 ± 0.06 x 0.49 ± 0.14 *(49,3)*	91	27	No tissue
Eurasian woodcock	Premaxilla	Multi-sided in cross section giving the bill-tips a 'honey-combed' appearance. 'Flat bottomed' with broad perforations communicating with underlying nerve channels	Distal-most face forwards, all others face outwards.	0.35 ± 0.07 x 0.37 ± 0.10 *(23,1)*	159	132	86
	Dentary	As above.		0.32 ± 0.05 x 0.36 ± 0.05 *(23,1)*	134	116	131
Bar-tailed godwit	Premaxilla	Oval in cross-section, tapering with depth. Broad, ovoid perforations at the base communicate with underlying nerve channels.	All pits forward-facing.	0.24 ± 0.05 x 0.33 ± 0.10 *(20,1)*	110	43	0
	Dentary	As above.		0.23 ± 0.04 x 0.39 ± 0.15 *(20,1)*	49	41	No tissue

Number of pits were counted from micro CT scans of segments of the bill bones between 0-5 mm; 5-10 mm and 10-15 mm proximal of the tip of the premaxilla and dentary bones.

The stilt and oystercatcher possessed small numbers of neurovascular foramina in the dentary (1 foramen; oystercatcher) and dentary and premaxilla (12 and 4 foramina, respectively; stilt), which were continuously connected with the major nerve channels. 

Herbst corpuscles were present within bill-tip sensory pits in the godwit and all kiwi specimens. They were arranged along the sides of the pits, surrounding central nerve fibers and blood vessels (Figure 3B, C). In sagittal sections, large nerves were also visible running within the godwit premaxilla and dentary, presumably contained in the bony channels described above. On average, 10.27 ± 5.59 Herbst corpuscles were visible per bill-tip sensory pit in the godwit (n = 15 sensory pits). Significantly fewer were visible per sensory pit in kiwi than in the godwit (6.32 ± 2.38 n = 38 pits, 4 kiwi; Kruskal Wallis H = 5.91, df = 1, p = 0.015). There were no significant differences in the number of Herbst corpuscles visible per pit among kiwi specimens (Mann-Whitney U-tests, all p > 0.05). 

### Brain allometry and morphology

**Figure 4:**
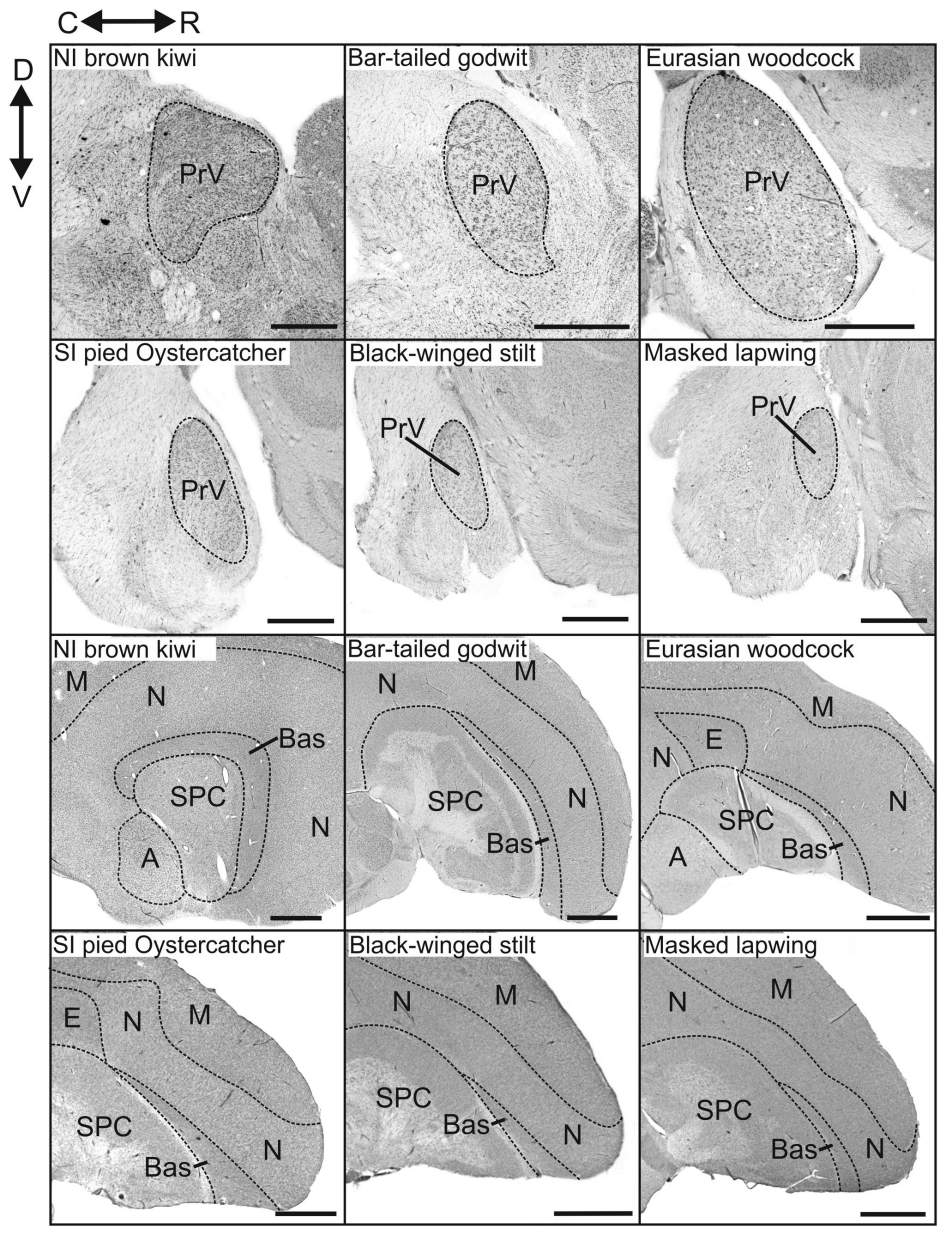
Sagittal sections of the brains of six species of birds examined in this study. Photomicrographs of sagittal sections stained with cresyl violet through the brain of six species of birds examined in this study. The top panel shows the principal sensory trigeminal nucleus (PrV) and the bottom panel the nucleus basorostralis (Bas). The broken black lines indicate the borders of each of the regions present in the sections. Brain sections are shown from North Island brown kiwi (*Apteryx mantelli*), bar-tailed godwit (*Limosa lapponica*), Eurasian woodcock (*Scolopax rusticola*), South Island oystercatcher (*Haematopus finschi*), black-winged stilt (*Himantopus himantopus*), and masked lapwing (*Vanellus miles*). Abbreviations: A: arcopallium, N: nidopallium, H: hyperpallium, E: entopallium, SPC: striatopallidal complex, M: mesopallium, C: caudal, R: rostral, D: dorsal, V: ventral. Scale bars; top panel = 1 mm, bottom panel = 2 mm.

**Figure 5:**
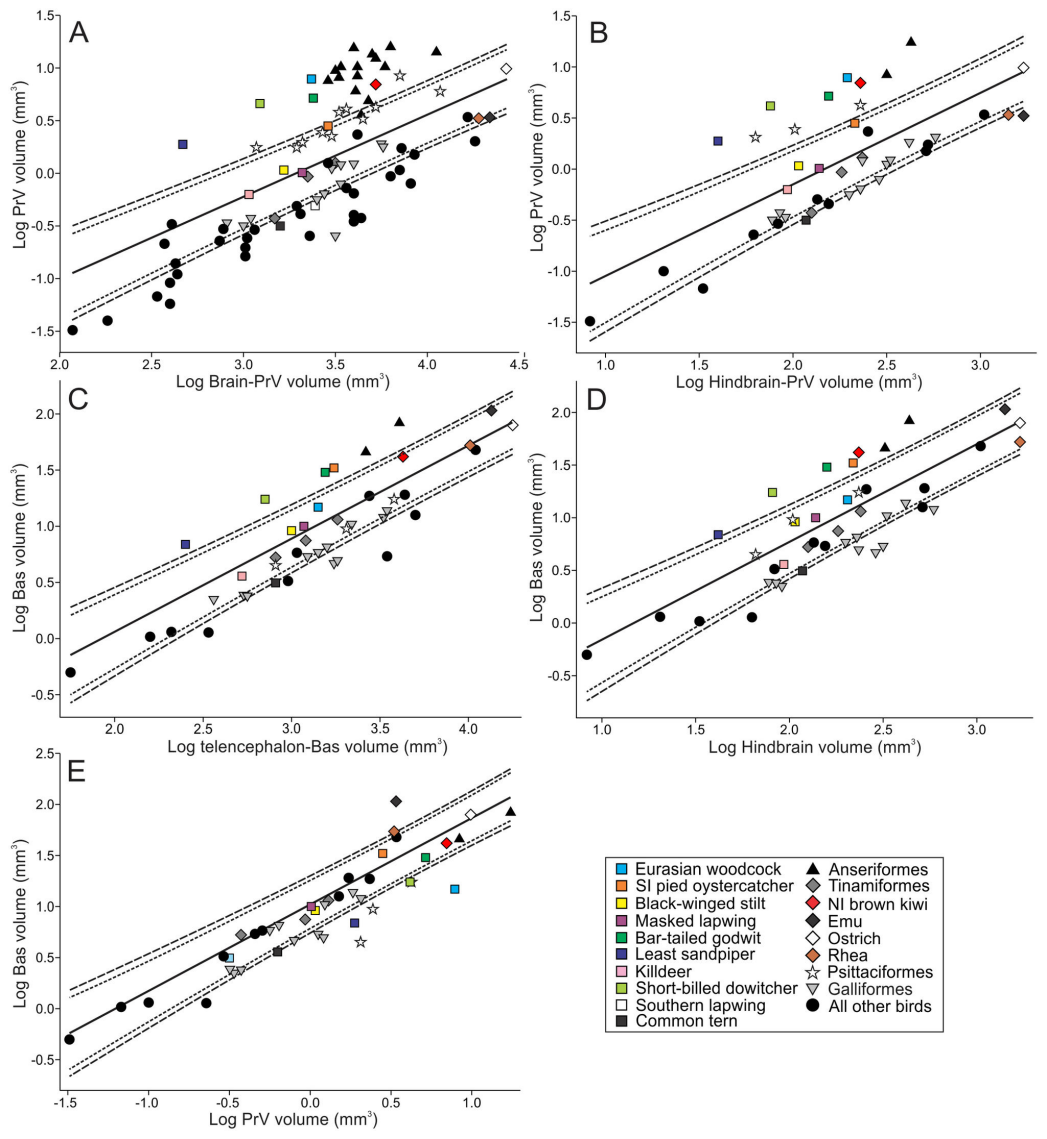
PrV and Bas volumes relative to the volume of the brain and hindbrain. Scatterplots of the log-volume of the principal sensory trigeminal nucleus (PrV) and the nucleus basorostralis (Bas, all measurements in mm^3^). Data from [8] and [37] is included in the analysis. PrV is regressed against A: Brain-PrV volume and B: hindbrain-PrV volume and Bas against C: telencephalon-Bas volume and D: hindbrain volume. E shows Bas regressed against PrV. The solid lines indicate the least-squares linear regression lines and the dotted and broken lines indicate the phylogeny-corrected 90 and 95% confidence interval, respectively. Symbols and colors represent birds and orders as shown in the legend.

In the dorsolateral brainstem, PrV was found at the level of the cerebellar peduncles in all species, and was clearly visible in Nissl stained material where it appeared as a round or oval nucleus that stained darker than surrounding areas (Figure 4). The cytoarchitecture of PrV varied considerably among the species examined in this study. In kiwi, godwits, and woodcocks, PrV appeared particularly well developed, consisting of a densely packed cell group with well-defined boundaries. The relative size of PrV, when regressed against the total brain-PrV and hindbrain volume, was highly variable amongst the species examined in this study (Figure 5A, B). The largest relative PrV sizes were found in waterfowl, with nearly all species having a PrV size that fell above the 95% CI criterion for hypertrophy. Parrots also had large relative PrV sizes with 4 species falling above the 95% CI criterion, and 7 species falling within this criterion. Conversely, galliforms had relatively small PrV sizes with some falling below the lower 95% CI bound. The shorebirds exhibited the greatest variability in relative PrV size of any order we examined. Woodcocks, godwits, least sandpipers (*Calidris minutilla*) and short-billed dowitchers (*Limnodromus griseus*) had a hypertrophied PrV, black-winged stilts, killdeer (*Charadrius vociferus*) and masked lapwings an intermediate (or average) PrV and common terns (*Sterna hirundo*) and Southern lapwings (*Vanellus chilensis*, [8]) a hypotrophied PrV (Figure 5A, B). The relative PrV volume in oystercatchers fell above the 90% CI criterion but within the 95% CI. In addition, the relative PrV volume in kiwi fell above the 95% CI criterion, unlike that of other paleognathous birds (including emus *Dromaius novahollandiae*, ostriches *Struthio camelus*, rheas *Rhea americana* and the 3 tinamou species), all of which had an intermediate or small relative PrV volume (Figure 5A, B). 

**Figure 6:**
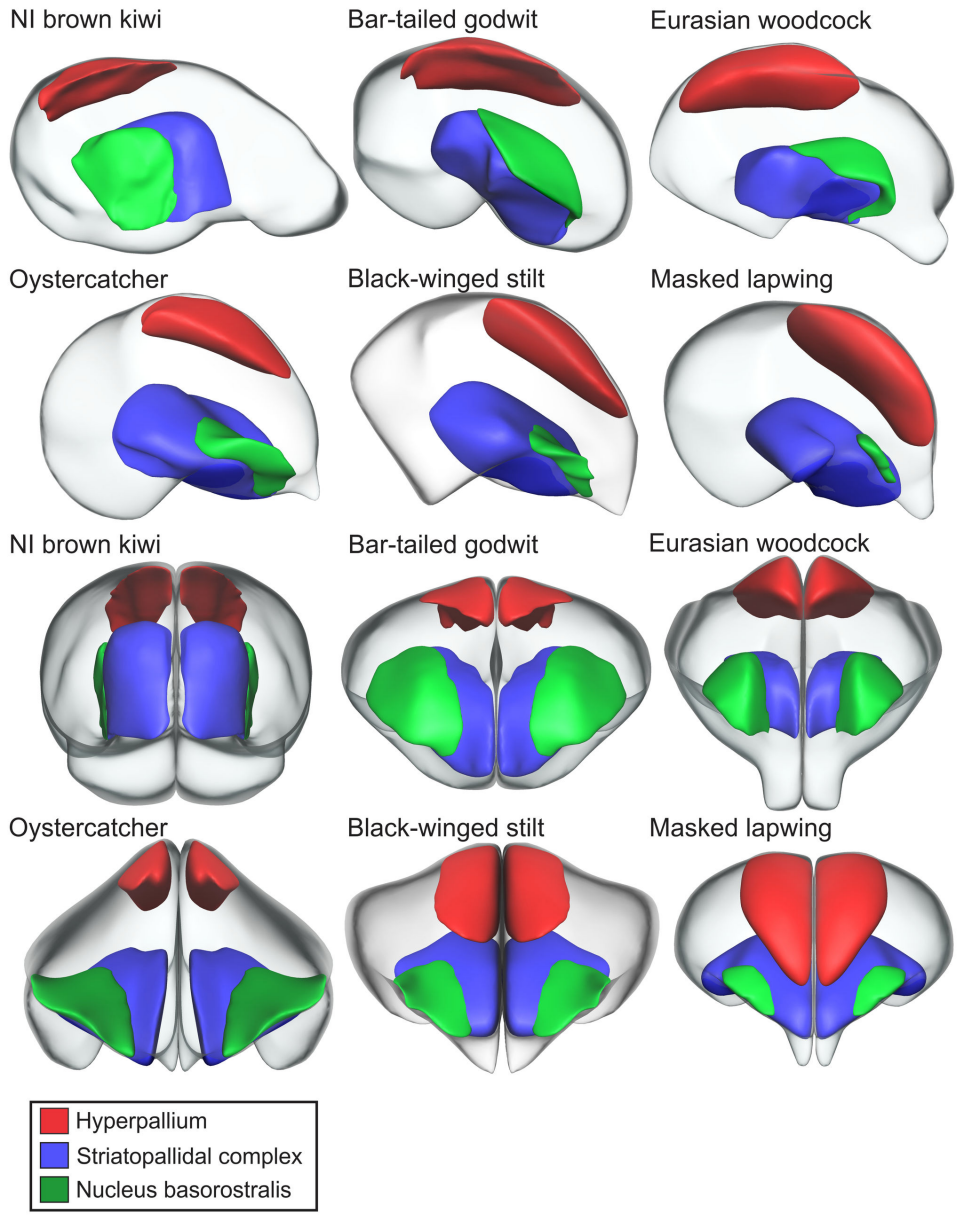
3D reconstructions of brain structures in six species of birds examined in this study. 3D reconstructions of the telencephalon (transparent), nucleus basorostralis (green), striatopallidal complex (blue) and the hyperpallium (red) in six species of birds. Models are shown in a lateral view in the top half of the panel and in a rostral view in the bottom half. Models are shown for North Island brown kiwi (*Apteryx mantelli*), bar-tailed godwit (*Limosa lapponica*), Eurasian woodcock (*Scolopax rusticola*), South Island oystercatcher (*Haematopus finschi*), black-winged stilt (*Himantopus himantopus*), and masked lapwing (*Vanellus miles*).

Nucleus basorostralis (Bas) was identified as a thin sheet of cells that surrounded the rostral face of the striatopallidal complex (SPC) in sagittal sections (Figure 4). The relative size of Bas, when regressed against the telencephalon-Bas and hindbrain volume, was generally hypertrophied in waterfowl and below average to hypotrophied in galliforms, which appear to mirror the size of PrV in these species (Figure 5C, D). In parrots, the relative sizes of Bas were generally average or below average. Of the shorebirds, godwits, oystercatchers, least sandpiper and short-billed dowitcher had a hypertrophied Bas, whereas common terns had a hypotrophied Bas. Kiwi had a hypertrophied Bas only when regressed against the hindbrain. The reason kiwi Bas was not hypertrophied when regressed against the telencephalon-Bas is probably because of the enlarged telencephalon in kiwi [43,44]. The architecture of Bas was similar in all species except that it appears to extend further dorsally in kiwi, godwits, and woodcocks (Figure 6). However, in kiwi, Bas is more laterally placed and extended further caudally than the other species examined in this study (Figure 6).

When Bas is regressed against PrV, a hypertrophy would suggest that Bas has enlarged but PrV has not mirrored this enlargement. Conversely, a hypotrophy suggests that PrV has enlarged but Bas has not paralleled this enlargement (Figure 5E). All parrots that were examined in this study showed a hypotrophy, suggesting that PrV was enlarged, but not Bas. Interestingly, of the shorebirds, woodcocks, together with least sandpipers, short-billed dowitchers and killdeer all had an enlarged PrV, but not an enlarged Bas. Conversely, emus and rheas were the only species to show a hypertrophy suggesting that Bas is enlarged, but not PrV (Figure 5E). 

From the 3D models of the telencephalon, it appears that the hyperpallium in kiwi, godwits and woodcocks is restricted to a caudal location and does not show the rostral extension found in the other birds examined (Figure 6). When comparing the location of the hyperpallium in oystercatchers, black-winged stilts and masked lapwings, the hyperpallium is most caudally placed in oystercatchers and most rostral in masked lapwings, with the hyperpallium in stilts in an intermediate position (Figure 6). 

## Discussion

We have shown that North Island brown kiwi have undergone an enlargement of the trigeminal system in a manner similar to shorebirds in the family Scolopacidae, providing evidence for parallel or convergent evolution of a complex tactile specialization across these phylogenetically disparate groups. Kiwi share with scolopacids a bill-tip organ consisting of mechanoreceptors clustered within sensory pits (as opposed to the touch papillae found in parrots and waterfowl, [1,21]), and a hypertrophied PrV. In addition, the Bas of kiwi is also hypertrophied, but not all of the scolopacids we examined showed a similar enlargement. Sample sizes for all species examined in this study were unavoidably small (1 - 4 individuals) and we therefore urge caution in interpretation of the patterns of morphology we found. Despite this, it seems unlikely that intra-specific variability in the anatomy of highly specialized sensory structures will often be greater than inter-specific variability. We therefore believe that the qualitative assessment of inter-specific differences in the trigeminal system that we present here is justified. 

### Somatosensory neural structures and the principle of proper mass

The large PrV and Bas in kiwi is probably the result of selective pressures towards the enhanced processing of tactile information from the bill, as is the case in species such as waterfowl and parrots [8,33,37]. This is also true for dunlins (*Calidris alpina*, Scolopacidae) where a tactile fovea in the frontal telencephalon (probably Bas) appears to be associated with increased trigeminal innervation from the receptors in the bill tip [79]. The enlargement of PrV and Bas in kiwi is also consistent with the suggestions of Martin, et al. [12] and Cunningham, et al. [11], and shown experimentally by Cunningham, et al. [80], that the bill tip of kiwi is specialized for the detection and localization of prey beneath the ground, as it is in Scolopacidae. The distant relationship between kiwi (Apterygiformes) and shorebirds (Charadriiformes) [27,28], suggests that hypertrophy of the PrV and Bas evolved independently in each group, hand-in-hand with their convergent evolution of a bill-tip organ capable of remote-touch foraging. Therefore, a hypertrophy of PrV and Bas is more likely correlated with enhanced sensory capabilities (principle of proper mass [41]) than any kind of phylogenetic ‘constraint’. 

A remarkable diversity of foraging strategies also exists among Charadriiformes, ranging from the bill-probing snipes to plunge-diving terns [10,81]. Even among ground-foraging shorebirds, the family Charadriidae contains species that hunt by vision, whilst the Scolopacidae are primarily tactile foragers, with each group having very different bill morphology and visual fields [82,83]. It is therefore unsurprising that this behavioral and ecological diversity is associated with a range of tactile receptor densities and relative PrV and Bas volumes. For instance, all Scolopacidae shorebirds we examined possessed a hypertrophied PrV, a feature shared with kiwi, waterfowl and parrots [8]. However, of the shorebirds from other families, only South Island oystercatchers (Haematopodidae) showed evidence of PrV enlargement, and PrV was hypotrophied in Southern lapwings (Charadriidae) and common terns (Laridae) – in keeping with the fact that the latter two species appear to rely primarily on vision for foraging rather than probe feeding with the bill [84,85]. It therefore appears that, within Charadriiformes, the enlargement of PrV reflects the degree of tactile specialization of the bill. 

The link between tactile foraging specializations and enlargement of Bas is much less clear than for PrV, despite the function of Bas as the telencephalic sensory end-station for trigeminal input [31-35]. Under the principle of proper mass [41], Bas should be hypertrophied in all species possessing a tactile bill-tip organ and hypertrophy of PrV. However, we found a hypertrophied Bas only in kiwi, waterfowl and some shorebirds. The explanation for this apparent discrepancy may lie in the multiple roles played by Bas in different species. For example, in addition to PrV input, Bas receives auditory input from the lateral lemniscus [31,86-91], and even vestibular input in a few species [31,90]. Furthermore, Bas differs in somatosensory representations among species, containing representations of the bill and cochlea in pigeons and finches [31,75-77], and in addition, a somatotopic representation of the whole body in budgerigars (*Melopsittacus undulatus*) and barn owls (*Tyto alba*) [78,91,92]. To a lesser extent, PrV also receives inputs from cranial nerves other than those from the trigeminal nerve in some species. For example, PrV receives glossopharyngeal input in waterfowl and hypoglossal input in seed eating species such as finches and budgerigars [5,29,62,63]. Therefore, the sizes of PrV and Bas could be influenced by other sensory information, which would lead to the size of these regions varying with sensory systems other than the trigeminal pathway. 

### Morphology of the bill tip in relation to foraging ecology and the trigeminal system

Bill-tip organs like those of kiwi, godwits and woodcocks are also found in other Scolopacidae species and in ibises [13,19]. In all of these avian families, the bill-tip organ is used to detect prey hidden in the substrate via substrate-borne vibrations [7,13,80], or via gradients in interstitial water pressure (in red knots *Calidris canutus* only, [9]) – an ability known as ‘remote touch’. Despite their broad overall similarity, bill-tip organs in bill-probing birds show differences in morphological detail, which seem to have functional significance. For example, in ibises the number and density of sensory pits reflects whether a species forages in granular substrates or water (where vibratory cues will travel very fast [13]). In scolopacids, the orientation of pits may depend on whether species use substrate-borne vibrations or interstitial pressure gradients to detect prey [9] and the number of mechanoreceptors within each pit seems to coincide with the degree of reliance on probe-foraging [19,20,24]. Indeed, it has been suggested that subtle differences in bill-tip organ morphology underlie niche differentiation in sandpipers [81]. 

We therefore speculate that the differences in bill-tip organ morphology we observed among kiwi, godwits and woodcocks, relate to their ecological niches and the demands these place on their somatosensory abilities. For example, kiwi and godwits had very similar numbers of sensory pits in the bill tips, but the godwit had significantly more mechanoreceptors per pit than the kiwi. Kiwi forage in granular substrates that are likely to be drier than those used by godwits, and use both remote-touch and olfaction when foraging [80]. If the number of mechanoreceptors per pit is indeed related to the degree of reliance on the remote-touch system [20], the use of multiple sensory systems by kiwi may explain the lesser numbers of mechanoreceptors in their bill. Woodcocks possess much higher numbers of sensory pits in the bill-tip than kiwi or godwits and these pits are flat-sided and closely packed. Woodcocks’ visual fields do not include the bill tip [82], and unlike kiwi, woodcocks are not known to possess a highly specialized sense of smell. Furthermore, woodcocks rely on probe-foraging year round, whereas godwits take invertebrates from the surface on their summer breeding grounds. A heavy dependence on tactile cues for foraging may therefore explain the much greater numbers and density of sensory pits in woodcock’s, than kiwi or godwit’s bill-tip organs. 

The differences in sensory pit structure, numbers and orientation between godwits and woodcocks are an example of the enormous variety of bill-tip organ morphologies found within the family Scolopacidae, further attested to in descriptions by [10,19,24] and [9]. Species within this family forage in a variety of habitats from mudflats and sandy beaches to marshes and woodland. Further study of the relationships between ecology, bill tip morphology and trigeminal specializations in this group is therefore likely to yield important information regarding the relationships between the structure and functioning of bill-tip organs in birds. 

In conclusion, the broad similarities we documented between kiwi and Scolopacidae bill-tip organs are likely to be a result of similar ecological selective pressures, while inter-specific differences in morphological detail may reflect fine-scale foraging-niche differentiation. Hypertrophy of PrV in these and other tactile-specialist orders of birds (e.g. parrots and waterfowl) appear to have co-evolved alongside bill tip specializations, in keeping with the principle of proper mass, whereas hypertrophy of Bas may be influenced to a greater extent by other sensory inputs. The bill-tip organ of bill-probing birds and the associated somatosensory regions in the brain subserve a remarkable ability to remote-detect prey in a manner likely to be far more efficient than probing by direct touch alone [20]. The success of this sensory system is attested to by its independent evolution in three avian lineages that are only distantly related: the Apterygidae, Threskiornithidae, and Scolopacidae. 

## Supporting Information

Table S1 **List of the species and volumes (in mm3) of their brain and the principal sensory trigeminal nucleus (PrV) obtained from Gutiérrez-Ibáñez [8] and the telencephalon (Tel), hindbrain (HB), and nucleus basorostralis (Bas) obtained from Boire** [37].
(DOCX)
