# Supplementary material for: The Anatomy of the bill Tip of Kiwi and Associated Somatosensory Regions of the Brain: Comparisons with Shorebirds
Source: PLoS One. 2013 Nov 14;8(11):e80036. doi: 10.1371/journal.pone.0080036 (PMC3828210; doi:10.1371/journal.pone.0080036)
Supplement: Table S1 — List of the species and volumes (in mm3) of their brain and the principal sensory trigeminal nucleus (PrV) obtained from Gutiérrez-Ibáñez [8] and the telencephalon (Tel), hindbrain (HB), and nucleus basorostralis (Bas) obtained from Boire [37]. (DOCX) [file pone.0080036.s001.docx]

Table S1. List of the species and volumes (in mm^3^) of their brain and the principal sensory trigeminal nucleus (PrV) obtained from Gutiérrez-Ibáñez [8] and the telencephalon (Tel), hindbrain (HB), and nucleus basorostralis (Bas) obtained from Boire [37].

| **Order** | **Common name** | **Species** | **Brain** | **Tel** | **HB** | **PrV** | **Bas** |
| --- | --- | --- | --- | --- | --- | --- | --- |
| Anseriformes | Green-winged teal | *Anas carolinensis* | 3165.83 | - | - | 9.43 | - |
|  | Chestnut teal | *Anas castanea* | 3424.71 | - | - | 10.14 | - |
|  | Northern shoveler | *Anas clypeata* | 3288.51 | - | - | 8.12 | - |
|  | Blue-winged teal | *Anas discors* | 2895.75 | - | - | 7.57 | - |
|  | Mallard | *Anas platyrhynchos* | 6343.98 | 4156.98 | 436.52 | 15.88 | 83.58 |
|  | Australian black duck | *Anas superciliosa* | 4973.94 | - | - | 13.50 | - |
|  | Lesser scaup | *Aythya affinis* | 4141.89 | - | - | 10.19 | - |
|  | Redhead | *Aythya americana* | 5245.17 | - | - | 12.19 | - |
|  | Canada goose | *Branta canadensis* | 11346.91 | - | - | 14.09 | - |
|  | Bufflehead | *Bucephala albeola* | 4122.97 | - | - | 6.05 | - |
|  | Common goldeneye | *Bucephala clangula* | 5961.39 | - | - | 10.15 | - |
|  | Australian wood duck | *Chenonetta jubata* | 4329.15 | - | - | 3.57 | - |
|  | Red-breasted merganser | *Mergus serrator* | 4754.34 | - | - | 4.87 | - |
|  | Ruddy duck | *Oyura jamaicensis* | 3993.73 | - | - | 15.64 | - |
| Apodiformes | Chimney swift | *Chaetura pelagica* | 342.66 | 159.92 | 33.11 | 0.07 | 1.04 |
| Caprimulgiformes | Nightjar | *Caprimulgus sp.* | 733.59 | 342.75 | 63.10 | 0.23 | 1.13 |
|  | Spotted nightjar | *Eurostopodus argus* | 1012.55 | - | - | 0.20 | - |
| Charadriiformes | Least sandpiper | *Calidris minutilla* | 472.01 | 255.50 | 41.69 | 1.89 | 6.91 |
|  | Killdeer | *Charadrius vociferus* | 1073.36 | 523.69 | 93.33 | 0.63 | 3.60 |
|  | Short-billed dowitcher | *Limnodromus griseus* | 1230.79 | 725.11 | 81.28 | 4.59 | 17.52 |
|  | Common tern | *Sterna hirundo* | 1592.66 | 808.53 | 117.49 | 0.32 | 3.13 |
|  | Southern lapwing | *Vanellus chilensis* | 2461.00 | - | - | 0.49 | - |
| Ciconiiformes | Grey heron | *Ardea cinerea* | 8445.95 | 5028.04 | 512.86 | 1.50 | 12.62 |
|  | Cattle egret | *Bubulcus ibis* | 4025.1 | - | - | 0.35 | - |
|  | Snowy egret | *Egretta thula* | 3610.00 | - | - | 0.72 | - |
| Columbiformes | Rock dove | *Columba livia* | 2219.55 | 1077.33 | 134.90 | 0.52 | 6.59 |
|  | Peaceful dove | *Geopelia placida* | 776.06 | - | - | 0.30 | - |
|  | Superb fruit-dove | *Ptilinopus superbus* | 1052.12 | - | - | 0.24 | - |
|  | Ringneck dove | *Streptopelia risoria* | 1140.93 | 950.83 | 83.18 | 0.29 | 3.25 |
| Coraciiformes | Laughing kookaburra | *Dacelo novaeguineae* | 3970.08 | - | - | 0.64 | - |
| Falconiformes | Swainson’s hawk | *Buteo swainsoni* | 8099.42 | - | - | 0.80 | - |
|  | American kestrel | *Falco sparverius* | 1017.00 | - | - | 0.16 | - |
| Galliformes | Chukar | *Alectoris chukar* | 2500.00 | 1406.39 | 199.53 | 0.56 | 5.86 |
|  | Ruffed grouse | *Bonasa umbellus* | 3146.72 | - | - | 0.26 | - |
|  | Golden pheasant | *Chrysolophus pictus* | 3368.73 | 1726.01 | 288.40 | 0.80 | 4.68 |
|  | Northern bobwhite | *Colinus virginianus* | 1090.73 | 569.85 | 85.11 | 0.37 | 2.40 |
|  | Common quail | *Coturnix coturnix* | 810.81 | 365.32 | 91.20 | 0.34 | 2.24 |
|  | Chicken | *Gallus domesticus* | 2993.00 | 1235.65 | 316.23 | 1.12 | 5.38 |
|  | Turkey | *Meleagris gallopavo* | 6096.95 | 3323.33 | 588.84 | 2.84 | 13.88 |
|  | Helmeted guineafowl | *Numida meleagris* | 3950.77 | 2223.28 | 331.13 | 1.23 | 10.47 |
|  | Chaco chachalaca | *Ortalis canicollis* | 3373.55 | 1829.65 | 234.42 | 1.21 | 4.96 |
|  | Ring-necked pheasant | *Phasianus colchicus* | 2761.58 | 1579.09 | 229.09 | 0.64 | 6.56 |
| Gruiformes | American coot | *Fulica americana* | 2875.00 | - | - | 1.25 | - |
|  | Red-gartered coot | *Fulica armillata* | 4015.00 | - | - | 0.40 | - |
| Passeriformes | Brown thornbill | *Acanthiza pusilla* | 434.36 | - | - | 0.11 | - |
|  | Eastern spinebill | *Acanthorhynchus tenuirostris* | 395.75 | - | - | 0.09 | - |
|  | Gouldian finch | *Erythrura gouldiae* | 427.61 | - | - | 0.14 | - |
|  | Australian magpie | *Gymnorhina tibicen* | 4017.37 | - | - | 0.31 | - |
|  | Noisy miner | *Manorina melanocephala* | 2278.96 | - | - | 0.25 | - |
|  | Spotted pardalote | *Pardalotus punctatus* | 400.58 | - | - | 0.06 | - |
|  | Double-barred finch | *Taeniopygia bichenovii* | 409.27 | - | - | 0.33 | - |
|  | Zebra finch | *Taeniopygia guttata* | 368.73 | 207.83 | 20.42 | 0.21 | 1.15 |
| Pelecaniformes | Double-crested cormorant | *Phalacrocorax auritus* | 7323.36 | 4341.73 | 524.81 | 1.73 | 19.03 |
| Podicipediformes | White-tufted grebe | *Rollandia rolland* | 2056.00 | - | - | 0.41 | - |
| Psittaciformes | Australian king parrot | *Alisterus scapularis* | 4478.76 | - | - | 3.27 | - |
|  | Long-billed corella | *Cacatua tenuirostris* | 11777.99 | - | - | 6.00 | - |
|  | Galah | *Eolophus roseicapillus* | 7083.98 | - | - | 8.40 | - |
|  | Purple-crowned lorikeet | *Glossopsitta porphyrocephala* | 1939.19 | - | - | 1.75 | - |
|  | Budgerigar | *Melopsittacus undulatus* | 1185.77 | 825.12 | 66.07 | 1.76 | 4.48 |
|  | Cockatiel | *Nymphicus hollandicus* | 2111.00 | - | - | 1.97 | - |
|  | Blue-headed parrot | *Pionus menstruus* | 5282.82 | 3851.82 | 234.42 | 4.23 | 17.24 |
|  | Crimson rosella | *Platycercus elegans* | 3628.38 | - | - | 4.08 | - |
|  | Superb parrot | *Polytelis swainsonii* | 2996.14 | - | - | 2.25 | - |
|  | Rainbow lorikeet | *Trichoglossus haematodus* | 3333.98 | - | - | 3.81 | - |
| Rheiformes | Greater rhea | *Rhea americana* | 19000.00 | 10281.31 | 1698.24 | 3.33 | 52.86 |
| Sphenisciformes | Magellanic penguin | *Spheniscus magellanicus* | 16756.76 | 10890.21 | 1047.13 | 3.41 | 47.36 |
| Strigiformes | Great horned owl | *Bubo virginianus* | 17994.21 | - | - | 2.01 | - |
|  | Boobook owl | *Ninox boobook* | 6338.8 | - | - | 0.94 | - |
|  | Barn owl | *Tyto alba* | 7142.86 | - | - | 1.08 | - |
| Tinamiformes | Red-winged tinamou | *Rhynchotus rufescens* | 3377.41 | 1971.68 | 239.88 | 1.62 | 13.48 |
| Trochiliformes | Anna’s hummingbird | *Calypte anna* | 183.88 | - | - | 0.04 | - |
|  | Blue-tailed emerald | *Chlorostilbon melisugus* | 118.73 | 56.17 | 8.30 | 0.03 | 0.50 |
